# Repeated-Doses Toxicity Study of the Essential Oil of *Hyptis martiusii* Benth. (Lamiaceae) in Swiss Mice

**DOI:** 10.1155/2013/856168

**Published:** 2013-09-10

**Authors:** Germana Freire Rocha Caldas, Alice Valença Araújo, Giwellington Silva Albuquerque, Jacinto da Costa Silva-Neto, João Henrique Costa-Silva, Irwin Rose Alencar de Menezes, Ana Cristina Lima Leite, José Galberto Martins da Costa, Almir Gonçalves Wanderley

**Affiliations:** ^1^Department of Pharmaceutical Sciences, Federal University of Pernambuco, 50740-521 Recife, PE, Brazil; ^2^Department of Physiology and Pharmacology, Federal University of Pernambuco, 50670-901 Recife, PE, Brazil; ^3^Department of Histology and Embryology, Federal University of Pernambuco, 50670-901 Recife, PE, Brazil; ^4^Department of Physical Education and Sport Sciences, Federal University of Pernambuco, Vitória de Santo Antão, 55608-680 Recife, PE, Brazil; ^5^Department of Biological Chemistry, Regional University of Cariri, 63105-000 Crato, CE, Brazil

## Abstract

*Hyptis martiusii* Benth. (Lamiaceae) is found in abundance in Northeastern Brazil where it is used in traditional medicine to treat gastric disorders. Since there are no studies reporting the toxicity and safety profile of this species, we investigated repeated-doses toxicity of the essential oil of *Hyptis martiusii* (EOHM). Swiss mice of both sexes were orally treated with EOHM (100 and 500 mg/kg) for 30 days, and biochemical, hematological, and morphological parameters were determined. No toxicity signs or deaths were recorded during the treatment with EOHM. The body weight gain was not affected, but there was an occasional variation in water and food consumption among mice of both sexes treated with both doses. The hematological and biochemical profiles did not show significant differences except for a decrease in the MCV and an increase in albumin, but these variations are within the limits described for the species. The microscopic analysis showed changes in liver, kidneys, lungs, and spleen; however, these changes do not have clinical relevance since they varied among the groups, including the control group. The results indicate that the treatment of repeated-doses with the essential oil of *Hyptis martiusii* showed low toxicity in mice.

## 1. Introduction

The genus *Hyptis* (Lamiaceae), comprising approximately 400 species distributed across a wide area from Southern United States to Argentina, has been widely studied from an ethnopharmacological, pharmacological, and chemical point of view, mainly owing to the diversity of bioactive constituents found in essential oils and extracts, which have interesting biological effects such as antimicrobial, anticancer, and insecticidal properties [1]. Some species of the *Hyptis* genus, such as *Hyptis suaveolens*, *Hyptis pectinata*, *Hyptis crenata,* and *Hyptis fruticosa*, are characterized by the presence of essential oils with important biological activities such as antiseptic [2], antifungal [3], antibacterial [4], anti-inflammatory [5], antinociceptive [6], and antiulcer properties [7, 8] among others.

*Hyptis martiusii* Benth., commonly known as “*cidreira*-*do*-*campo*” or “*cidreira*-*brava,*” is an aromatic plant found in abundance in Northeastern Brazil and is characterized as a potential source of essential oils, like other species of the *Hyptis* genus. In folk medicine, the infusion or decoction of *Hyptis martiusii* leaves is used to combat intestinal and stomach diseases, while the decoction of the roots is used to counter inflammation of the ovaries [9]. Few studies have been carried out into the biological and pharmacological properties of *Hyptis martiusii*. Cytotoxic and antiproliferative effects on certain tumor cell lines [10, 11], insecticidal activity against the larvae of *Aedes aegypti* and *Culex quinquefasciatus* [12], antimicrobial activity against resistant strains of *Staphylococcus aureus* and *Escherichia coli* [13], and antioxidant activity [14] have been reported for *Hyptis martiusii*.

 Recently, our research group first reported that the essential oil of the leaves of *Hyptis martiusii* has an antiulcerogenic and antisecretory activity in acute gastric ulcer models [15]. Given that there are no studies regarding the toxicological profile of this species, a repeated-doses (30 days) toxicity study was conducted to evaluate the safety of the oral administration of the essential oil of *Hyptis martiusii*.

## 2. Material and Methods

### 2.1. Plant Material and Extraction of Essential Oil

*Hyptis martiusii* (Lamiaceae) leaves were collected on the Araripe Plateau in Crato in the Brazilian State of Ceará (S 7°21.744′-W 39°28.691′). Entire plants were collected during the flowering stage, in June 2011. A representative sample of this species is deposited in the Prisco Bezerra Herbarium of the Department of Biology at the Federal University of Ceará (UFC) (Registration no. 43038).

The leaves were dried at room temperature for 72 h prior to hydrodistillation and the essential oil was extracted immediately thereafter. Two portions (830.00 ± 30.00 g) of the dried leaves were individually subjected to hydrodistillation using a Clevenger-type apparatus for 3 h. The yield of the essential oil from dried leaves of *Hyptis martiusii* (EOHM) was 0.95 ± 0.03% (w/w), corresponding to 7.95 ± 0.55 g of oil, calculated according to the mean dry weight of the leaves used in each extraction. The water/oil mixture was collected, the aqueous solution was discarded, and the oil was dried over anhydrous sodium sulfate and then filtered. Essential oil was stored in an amber bottle at −20°C until the toxicological experiments and phytochemical analysis had been carried out.

### 2.2. Chemical Analysis of the Essential Oil

Analysis of the EOHM was performed using a gas chromatograph attached to a mass spectrometer (GC-MS, SHIMADZU QP5050A) equipped with a capillary column (DB-5HT, 30 m × 0.25 mm, 0.1 *μ*m-thick film) with the following specifications: helium as carrier gas (1.0 mL/min flow rate); injector temperature 270°C and detector temperature 290°C; a linear velocity of 47.3 cm/sec; a pressure of 107.8 kPa; a column temperature programmed to increase from 60°C (2 min) to 180°C (1 min) at 4°C/min and then from 180 to 260°C at 10°C/min (10 min). The mass spectrometer was operated using 70 eV of ionization energy. Identification of individual constituents was based on the interpretation of their mass spectral fragmentation using computer-based library MS search standards (Wiley 229), retention indices, and comparison with the mass spectra database and data from the literature [16].

### 2.3. Animals

All the experiments were conducted in accordance with the National Institute of Health's Guide for the Care and Use of Laboratory Animals and were submitted to and approved by the Animal Experimentation Ethics Committee of the UFPE (License no. 012490). Male and female Swiss mice (35–45 g) obtained from the Keizo Asami Immunopathology Laboratory (LIKA/UFPE, Pernambuco Brazil) were used in experiments after a one-week adaptation period in the laboratory. They were kept under standard environmental conditions (12 h dark/light cycle; temperature 22 ± 2°C). Water and industrialized dry food (Labina, Purina, Brazil) were made available *ad libitum*.

### 2.4. Repeated-Doses Toxicity Study

Healthy male and female Swiss mice were randomly divided into three groups (*n* = 10/group/sex). The essential oil of *Hyptis martiusii* was emulsified in a Tween-80 at 1% before administration to the animals; thus, the animals of control group received a Tween-80 1% aqueous solution and the treated groups received EOHM at doses of 100 and 500 mg/kg by oral route for 30 consecutive days. During the treatment, the body weight was recorded weekly, and food consumption and water intake of the animals were recorded every two days. Animals were observed twice daily for signs of toxicity, such as piloerection, diarrhea, and changes in locomotor activity, and mortality throughout the experimental period. At the end of the 30-day treatment, the animals were fasted overnight, although water was made available *ad libitum*. They were then anesthetized with thiopental (35 mg/kg, i.p.), and blood samples were obtained by retro-orbital puncture using capillary tubes and collected in two tubes: one tube containing the anticoagulant ethylenediaminetetraacetic acid (EDTA) and one tube without anticoagulant for hematological and biochemical parameters, respectively.

### 2.5. Hematological and Biochemical Analyses

Hematological analyses were carried out immediately after collection using an automatic hematology analyzer (Coulter STKS, Beckman). Parameters included red blood cell (RBC) count, white blood cell (WBC) count, hemoglobin (Hb), hematocrit (Hct), mean corpuscular volume (MCV), mean corpuscular hemoglobin (MCH), mean corpuscular hemoglobin concentration (MCHC), red cell distribution width (RDW), platelet count, mean platelet volume (MPV), and differential leukocyte count (lymphocytes, monocytes, neutrophils, eosinophils, and basophils). For biochemical analysis, blood was centrifuged at 1480 ×g for 10 min to obtain serum, which was stored at −20°C, and the following parameters were determined: glucose, blood urea nitrogen (BUN), creatinine, aspartate aminotransferase (AST), alanine aminotransferase (ALT), total cholesterol, triglycerides, total protein, albumin, and lactate dehydrogenase (HDL) [17]. Dosages were made using Architect (Abbott) automation with Boehringer Ingelheim biochemical kits.

### 2.6. Morphological Study

After blood collection, the animals (*n* = 5/group/sex) were euthanized with an excess of thiopental (140 mg/kg, i.p.) and a necropsy was performed for macroscopic external evaluation of the heart, lungs, liver, kidneys, adrenal glands, spleen, stomach, intestine, pancreas, brain, and reproductive organs (testicles and prostate (male) or uterus and ovaries (female)). These organs were carefully removed and weighed individually. Organ weights were expressed in absolute and relative terms (g and g/10 g of body weight, resp.).

For microscopic analysis, the remaining animals (*n* = 5/group/sex) were anesthetized, perfused with saline (to remove blood), and then the organs described previously were removed and fixed “*in totum*” in 10% buffered formalin for 48 h at room temperature. After fixing, each sample was washed with water and immersed in 70% ethyl alcohol for 3 to 4 days and then was embedded in paraffin. Paraffin sections of 5 *μ*m were obtained and stained with hematoxylin/eosin (HE) [18]. Histological analyses of organs were made using an automatic microscopy system MICRO DIP (Kacil Inc.).

### 2.7. Statistical Analysis

The results were expressed as mean ± standard error of mean (S.E.M). The differences among the groups were determined by one-way analysis of variance (ANOVA) followed by Dunnett's test. Statistical analysis was performed using GraphPad Prism 5.0. The level of significance for rejection of the null hypothesis was set at 5% (*P* < 0.05).

## 3. Results

### 3.1. Chemical Analysis of Essential Oil

The chemical characterization of the essential oil using GC-MS identified 27 components, accounting for 95.52% of the total oil. The major components identified were 1,8-cineole (32.80%), *δ*-3-carene (17.43%), camphor (6.70%), *α*-pinene (3.52%), and caryophyllene oxide (3.50%).  Table 1 shows the constituents identified, the percentage composition, and retention index (RI).

### 3.2. Repeated-Doses Toxicity Study

**Figure 1:**
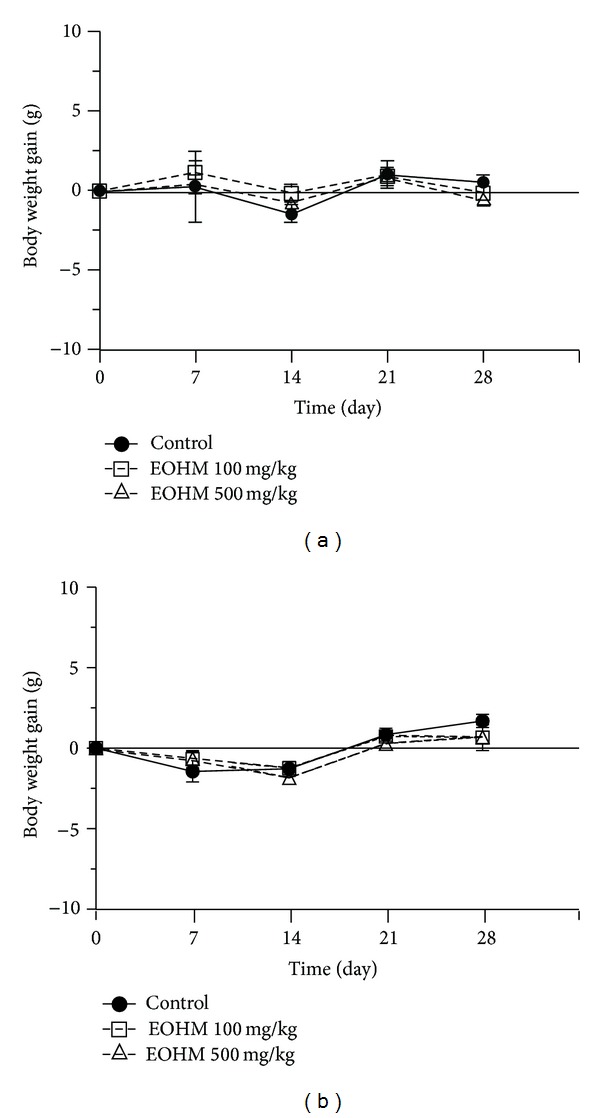
Effect of the essential oil of *Hyptis martiusii* (EOHM, 100 and 500 mg/kg, p.o.) on body weight gain (g) from male (a) and female (b) Swiss mice treated orally for 30 days. Values are expressed as mean ± S.E.M. (*n* = 10/group).

**Figure 2:**
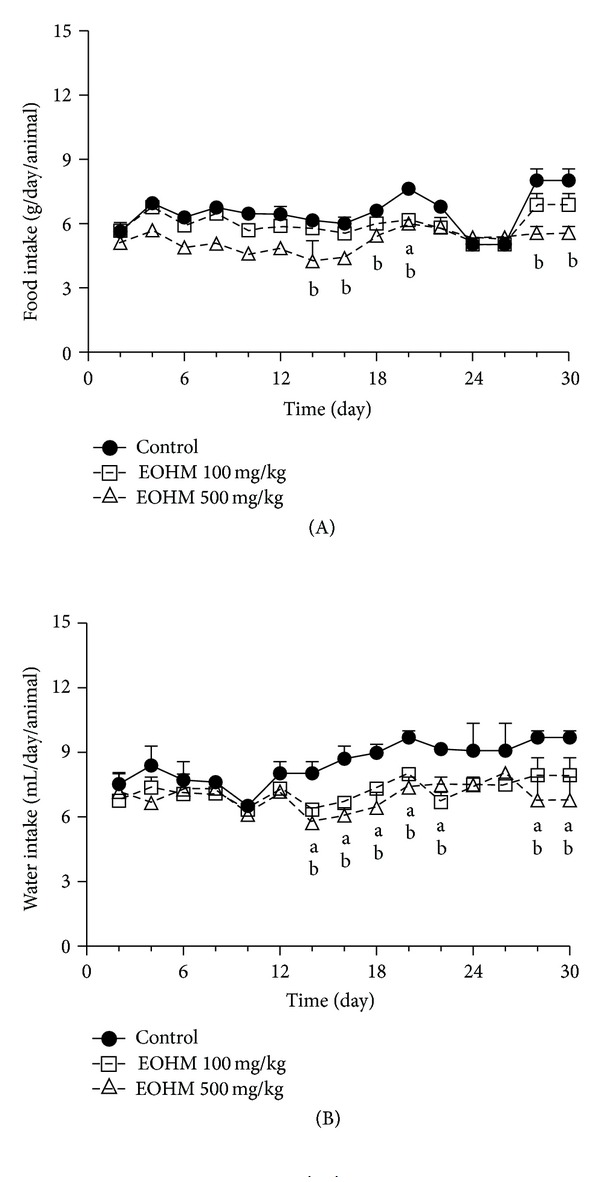
Effect of the essential oil of *Hyptis martiusii* (EOHM) on food (A) and water (B) intake in male Swiss mice treated orally for 30 days. Data are expressed as mean ± S.E.M. (*n* = 10/group), and letters represent differences in relation to the control group (a: EOHM 100 mg/kg and b: EOHM 500 mg/kg) at the same day (ANOVA followed by Dunnett's test, *P* < 0.05).

**Figure 3:**
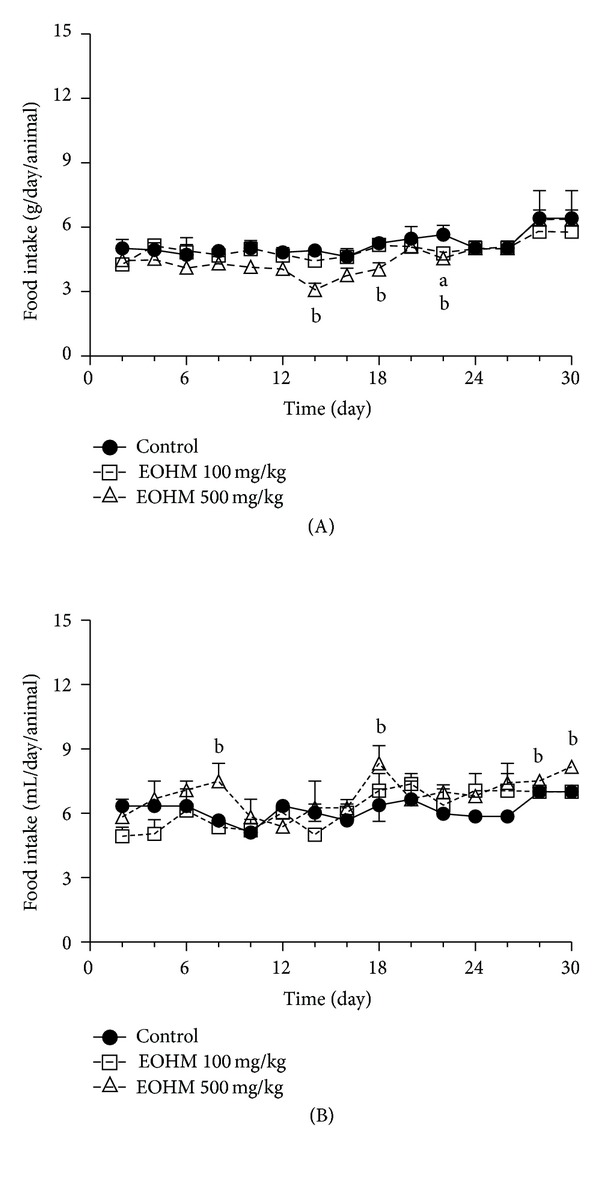
Effect of the essential oil of *Hyptis martiusii* (EOHM) on food (A) and water (B) intake in female Swiss mice treated orally for 30 days. Data are expressed as mean ± S.E.M. (*n* = 10/group), and letters represent differences in relation to the control group (a: EOHM 100 mg/kg and b: EOHM 500 mg/kg) at the same day (ANOVA followed by Dunnett's test, *P* < 0.05).

No signs of toxicity, such as piloerection, diarrhea, sedation, abdominal contortions, and alterations in locomotor activity or deaths, were recorded during the 30 consecutive days of treatment by oral route with the essential oil of *Hyptis martiusii* at the doses of 100 and 500 mg/kg. The body weight gain in mice of both sexes was not affected during the treatment with EOHM when compared to that of the mice in the control group (Figure 1). Occasional alterations in the food and water intake were observed in mice of both sexes during the treatment period in relation to the control group. There was a decrease in food and water intake in male mice treated with both doses (Figure 2) and a decrease in food intake and an increase in water intake in female mice treated likewise (Figure 3).

### 3.3. Hematological and Biochemical Parameters

The hematological profiles of the experimental and control groups are shown in Table 2. There were no changes in the hematological profile of groups treated with the essential oil of *Hyptis martiusii* (EOHM, 100 and 500 mg/kg) in either sex, except for a statistical decrease of 4.7% in the mean corpuscular volume (MCV) of male mice (EOHM 500 mg/kg) in relation to the control group. Regarding the biochemical parameters, in the group of male mice, no statistically significant differences were recorded for any of the parameters examined. In female mice, however, treatment with EOHM 500 mg/kg showed a significant increase of 24.1% in albumin when compared to that of the mice in the control group. The biochemical profile is shown in Table 3.

### 3.4. Morphological Parameters

The absolute and relative weights of the tissues were not changed by the treatment with *Hyptis martiusii*, except for an increase in relative weight of the kidneys (26.0%) of female mice treated with dose 500 mg/kg and a decrease in relative weight of the spleen (26.4%) of female mice treated with doses of 100 mg/kg (Table 4). The macroscopic analysis of target organs of the animals treated with essential oil of *Hyptis martiusii* did not show significant changes in color or texture when compared to that of the control group.

**Figure 4:**
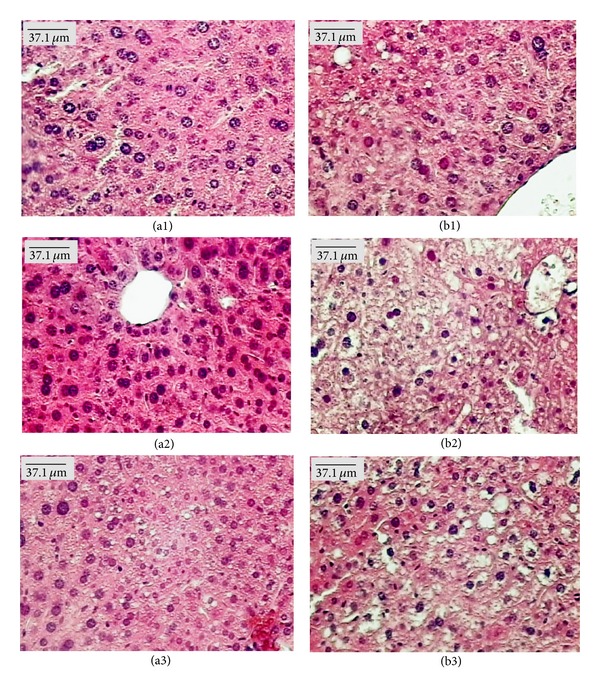
Paraffin sections of liver (HE) from male (a) and female (b) Swiss mice treated orally for 30 days with Tween-80 1% aqueous solution (Control, (a1) and (b1)) and essential oil of *Hyptis martiusii* (EOHM, 100 mg/kg ((a2), (b2)) and 500 mg/kg ((a3), (b3))).

**Figure 5:**
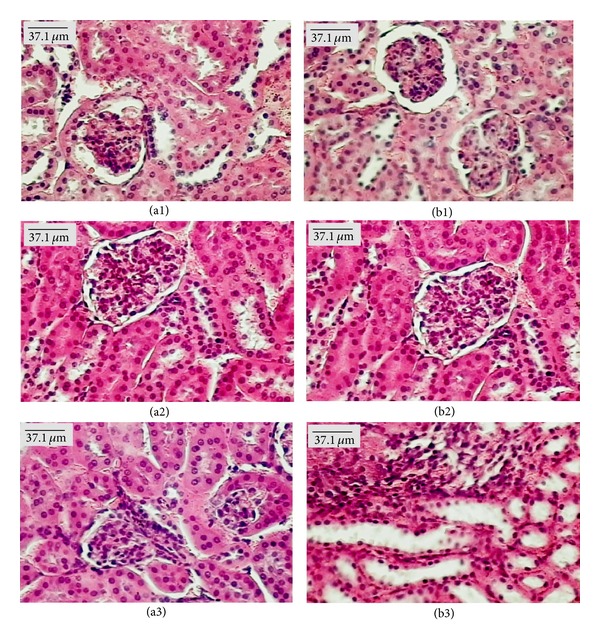
Paraffin sections of kidney (HE) from male (a) and female (b) Swiss mice treated orally for 30 days with Tween-80 1% aqueous solution (Control, (a1) and (b1)) and essential oil of *Hyptis martiusii* (EOHM, 100 mg/kg ((a2), (b2)) and 500 mg/kg ((a3), (b3))).

**Figure 6:**
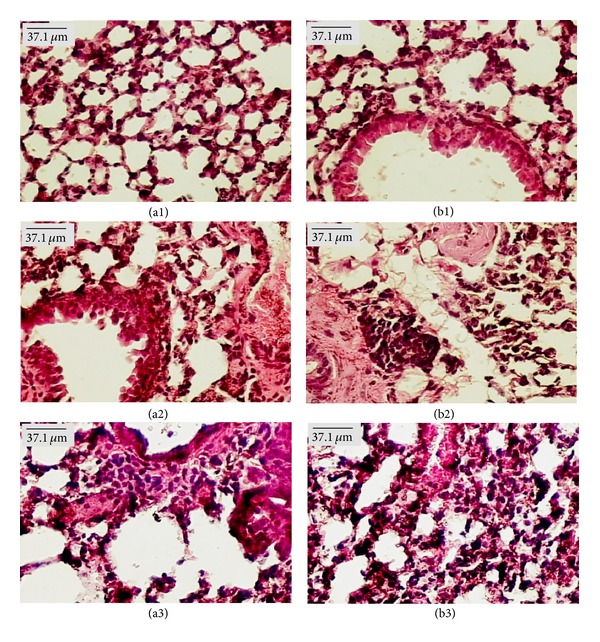
Paraffin sections of lung (HE) from male (a) and female (b) Swiss mice treated orally for 30 days with Tween-80 1% aqueous solution (Control, (a1) and (b1)) and essential oil of *Hyptis martiusii* (EOHM, 100 mg/kg ((a2), (b2)) and 500 mg/kg ((a3), (b3))).

**Figure 7:**
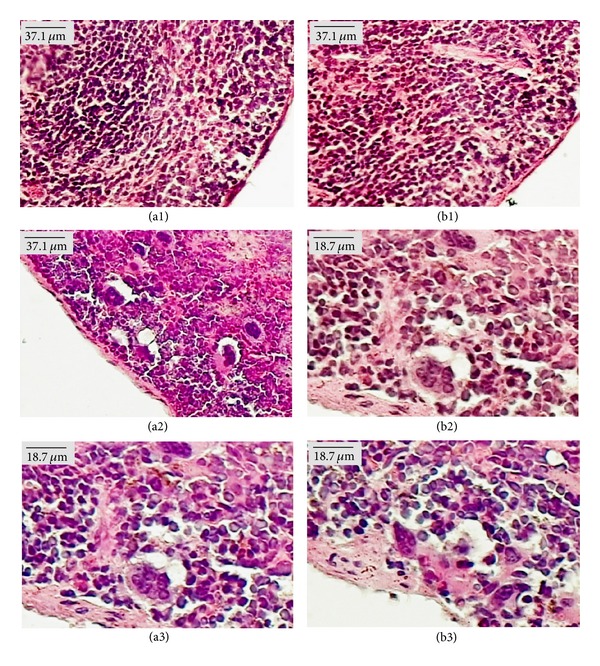
Paraffin sections of spleen (HE) from male (a) and female (b) Swiss mice treated orally for 30 days with Tween-80 1% aqueous solution (Control, (a1) and (b1)) and essential oil of *Hyptis martiusii* (EOHM, 100 mg/kg ((a2), (b2)) and 500 mg/kg ((a3), (b3))).

Microscopic examination of organs showed the presence of fat in the liver of females from the control group treated with both doses of EOHM, but it was not observed in the livers of male mice (Figure 4). A slight lymphocytic infiltrate was observed in the kidneys of females treated with EOHM (500 mg/kg) (Figure 5) and in the lungs of males and females treated with both doses of EOHM (Figure 6), as well as a slight increase in phagocytic activity (increase in number of macrophages) in the spleens of males and females treated with both doses of EOHM (Figure 7). The other organs of male and female mice in the experimental and control groups exhibited no histological alteration.

## 4. Discussion

The gastroprotective activity of the essential oil from the leaves of *Hyptis martiusii* was shown recently by our research group [15], confirming its ethnomedicinal use. In this study, we have assessed for the first time the repeated doses toxicity of the essential oil of the leaves of *Hyptis martiusii* (EOHM).

Few studies have reported the toxicity profile of the essential oils of the *Hyptis* genus. Raymundo et al. [19] and Menezes et al. [6] described the low acute toxicity of the essential oil of the leaves of *H*. *pectinata* and *H*. *fruticosa,* respectively, finding no deaths or any signs of toxicity up to a dose of 3 or 5 g/kg. Considering this scenery of little toxicological information available in the literature, this study was designed to obtain information regarding the safety profile of this species.

Our research group has recently demonstrated that, when administered orally at a dose of 5 g/kg, the essential oil of *Hyptis martiusii* brought on depression of the central nervous system (sedation) in mice of both sexes during the first 30 min and for a period of up to 4 h after administration. However, it produced no signs of acute toxicity or death in the treated animals, and no significant changes in food and water consumption or in body weight were observed during the 14 days of observation, suggesting a lethal dose (LD_50_, median lethal dose) above 5000 mg/kg, which is a characteristic of low toxicity [15].

IN a general way, the main constituents of essential oils are monoterpenes and sesquiterpenes, and this is also true of species of the *Hyptis* genus, in which these constituents have been identified as the main components [1]. In this study, GC-MS analysis of the essential oil of *Hyptis martiusii* identified 27 components and the main monoterpene and sesquiterpene components were 1,8-cineole, *δ*-3-carene, camphor, *α*-pinene, and caryophyllene oxide.

Oral administration at repeated-doses (30 days) of the essential oil of *Hyptis martiusii* in mice of both sexes did not cause death or any clinical signs of toxicity. There was an occasional variation in water and food consumption among mice of both sexes, but these variations did not influence the body weight of the animals during the treatment period, suggesting an absence of toxic effect. The doses used in this study represent the effective dose (100 mg/kg) and one dose five times higher (500 mg/kg). In studies of repeated dose toxicity, body weight gain and organ weight are considered important parameters and changes in them can indicate a toxic effect of the drug [20].

With the exception of the decrease in mean corpuscular volume (MCV) in male animals treated with EOHM (500 mg/kg), although levels are in the physiological limits described for the species [21], not an other hematological parameter was changed. These data indicate that the essential oil of *Hyptis martiusii* had no effects on the circulating blood cells or on their production. The analysis of blood parameters is important for risk evaluation, as any changes in the hematological system have a higher predictive value for human toxicity when data are translated from animal studies [22].

 In the analysis of biochemical parameters, no significant differences were found in serum levels of urea, creatinine, AST, ALT, glucose, cholesterol, triglycerides, and total protein among groups of both sexes treated with *Hyptis martiusii*. Only female mice treated with EOHM (500 mg/kg) had a slight increase in the level of albumin when compared to the control group. However, the value remained within the physiological limits described for the species [23]. The increase of plasmatic levels of BUN and creatinine is indicative of renal overload, acute renal failure, or increase in protein catabolism [24]. Moreover, the increase of serum transaminase enzymes (ALT and AST) is a good indicative of hepatocyte damage because both enzymes are found in higher concentrations in those cells and could be elevated due to impairment in transmembrane permeability or cellular damage [25]. Since the enzyme AST is also found in a large number of tissues, such as heart, lung, skeletal muscle, and kidney, whereas ALT is primarily limited to hepatocytes, the latter is considered a highly sensitive indicator of hepatotoxicity [26]. Therefore, the fact that the administration of *Hyptis martiusii* did not produce changes in these biomarkers suggests absence of renal and hepatic toxicity.

No abnormal signs were found in internal organs on macroscopic examination in animals treated with EOHM. Only the females presented an increase in the kidney relative weight and a decrease in the spleen relative weight. Since these changes were not found in males and there was no dose dependency, we attribute this change to a floating point not correlated to the treatment with essential oil of *Hyptis martiusii* and therefore it does not have clinical relevance.

Although fat was found in the liver and discrete lymphocytic infiltrate in the kidneys of females treated with EOHM, no adverse effect on the usual biomarkers of liver and kidney toxicity (liver enzymes, ALT, AST, and creatinine) was observed, suggesting that EOHM did not cause significant damage to these organs. As a slight lymphocytic infiltrate was observed in the kidneys of females treated with EOHM (500 mg/kg), it is possible that the kidney's relative weight increase observed could be due to an inflammatory edema. The occurrence of lymphocytic infiltrate observed in the lungs could be related to possible oil aspiration at the time of orogastric gavage.

Histopathological findings from the liver, kidneys, lungs, and spleen varied among mice of both sexes, even in the control group, and showed no correlation with the dose employed. Furthermore, the morphology of all other organs analyzed remained unchanged. Similar results have been found by Attawish et al. [27], who described the toxicity profile of repeated doses of *Hyptis suaveolens* in rats. These authors showed that animals treated with a dose of 500 mg/kg showed some alterations in tissue samples, such as fatty liver, myocarditis, nephrocalcinosis, pyelonephritis, splenomegaly, and granuloma sperm. However, this result was not widespread, and such changes were observed even in the control group.

## 5. Conclusions

 The data suggest that oral administration of the essential oil of *Hyptis martiusii* showed low toxicity in mice. The histopathological changes showed no clinically relevant changes, since they occurred in a non-generalized fashion in animals of the treated and control groups. However, it should be noted that further studies involving chronic toxicity, reproductive toxicity, genotoxicity, and carcinogenicity in other species (rodent and nonrodent) are needed to better assess the safety profile of *Hyptis martiusii*.

## Figures and Tables

**Table 1:** Chemical constituents of essential oil of leaves of *Hyptis martiusii* Benth.

Components	Retention time (min)	(%)
Hexen-1-ol	4.39	1.81
*α*-Pinene	6.59	3.52
*β*-Pinene	8.18	2.28
*β*-Myrcene	8.69	1.81
*δ*-3-Carene	9.60	17.43
*p*-Cymene	10.01	0.87
*o*-Cymene	10.35	3.36
1,8-Cineole	10.76	32.80
Linalool	13.89	1.21
Camphor	16.31	6.70
Isoborneol	17.54	0.99
trans-Caryophyllene	30.69	3.37
Aromadendrene	31.63	1.96
*α*-Humulene	32.51	1.69
Ledene	34.32	0.99
Germacrene B	34.59	2.21
*γ*-Selinen	36.58	0.77
*β*-Panasinsene	36.81	0.97
Isolongifol	37.74	0.97
Palustrol	38.71	0.83
Spathulenol	38.62	1.85
Caryophyllene oxide	38.82	3.50
Globulol	38.98	0.91
Ledol	39.85	1.04
Rosifoliol	40.04	0.86
(*Z*)-Valerenyl acetate	42.43	0.82
Monoterpenes hydrocarbons		29.27
Oxygenated monoterpenes		41.70
Sesquiterpenes hydrocarbons		11.96
Oxygenated sesquiterpenes		10.78
Others		1.81

Total identified		95.52

**Table 2:** Effect of the essential oil of *Hyptis martiusii* (EOHM, 100 and 500 mg/kg) on hematological parameters in male and female Swiss mice treated orally for 30 days.

Parameter	Control		EOHM 100 mg/kg		EOHM 500 mg/kg	
	Male	Female	Male	Female	Male	Female
Erythrocytes (10^6^/*µ*L)	9.59 ± 0.18	9.90 ± 0.10	9.40 ± 0.58	9.69 ± 0.17	8.96 ± 0.57	10.02 ± 0.17
Hemoglobin (g/dL)	16.40 ± 0.48	16.71 ± 0.33	16.51 ± 0.68	16.89 ± 0.15	14.64 ± 1.28	17.50 ± 0.22
Hematocrit (%)	45.34 ± 1.15	46.61 ± 0.28	44.26 ± 2.61	46.43 ± 0.45	40.06 ± 2.96	47.16 ± 0.90
MCV (fL)	47.71 ± 0.28	48.11 ± 1.03	47.57 ± 0.36	48.29 ± 0.35	45.43 ± 0.86*	47.71 ± 0.52
MCH (pg)	17.21 ± 0.09	17.24 ± 0.09	17.14 ± 0.25	17.44 ± 0.17	16.27 ± 0.54	17.30 ± 0.15
MCHC (g/dL)	36.17 ± 0.22	36.60 ± 0.10	36.60 ± 0.28	36.33 ± 0.14	35.93 ± 0.58	36.74 ± 0.10
RDW (%)	18.17 ± 0.48	15.53 ± 0.29	17.19 ± 0.40	15.47 ± 0.21	18.76 ± 1.08	15.59 ± 0.34
WBC (10^3^/*µ*L)	10.70 ± 1.03	12.36 ± 1.55	14.41 ± 1.26	11.70 ± 1.21	14.83 ± 2.04	13.67 ± 1.26
Platelets (10^3^/*µ*L)	1061.00 ± 98.70	872.60 ± 17.88	839.50 ± 91.68	761.70 ± 31.97	1140.00 ± 58.77	975.30 ± 73.71
MPV (fL)	6.07 ± 0.12	5.82 ± 0.13	6.00 ± 0.13	5.71 ± 0.09	6.31 ± 0.18	5.71 ± 0.09
Lymphocytes (%)	92.59 ± 0.70	94.53 ± 0.43	91.41 ± 0.64	92.83 ± 0.46	91.33 ± 0.80	92.89 ± 0.80
Monocytes (%)	0.87 ± 0.08	0.52 ± 0.06	0.75 ± 0.13	0.58 ± 0.09	1.61 ± 0.48	0.50 ± 0.06
Neutrophils (%)	6.32 ± 0.60	4.58 ± 0.41	7.05 ± 0.40	5.21 ± 0.46	6.45 ± 0.30	5.25 ± 0.64
Eosinophils (%)	0.05 ± 0.02	0	0.04 ± 0.02	0.05 ± 0.02	0.04 ± 0.04	0.04 ± 0.02
Basophils (%)	0.43 ± 0.06	0.35 ± 0.08	0.50 ± 0.03	0.45 ± 0.03	0.53 ± 0.16	0.41 ± 0.05

MCV: mean corpuscular volume, MCH: mean corpuscular hemoglobin, MCHC: mean corpuscular hemoglobin concentration, RDW: red cell distribution width, WBC: white blood cell, and MPV: mean platelet volume. Values represent the mean ± S.E.M (*n* = 10/group). *Statistically different from the control group (ANOVA followed by Dunnett's test, *P* < 0.05).

**Table 3:** Effect of the essential oil of *Hyptis martiusii* (EOHM, 100 and 500 mg/kg) on biochemical parameters in male and female Swiss mice treated orally for 30 days.

Parameter	Control		EOHM 100 mg/kg		EOHM 500 mg/kg	
	Male	Female	Male	Female	Male	Female
Glucose (mg/dL)	105.20 ± 4.70	104.40 ± 5.46	88.57 ± 6.62	98.00 ± 13.95	107.40 ± 4.62	91.33 ± 9.46
BUN (mg/dL)	47.17 ± 4.44	41.41 ± 4.99	49.17 ± 5.52	49.68 ± 2.36	48.87 ± 3.41	34.83 ± 3.53
Creatinine (mg/dL)	0.22 ± 0.03	0.21 ± 0.02	0.24 ± 0.04	0.22 ± 0.03	0.28 ± 0.02	0.22 ± 0.02
AST (U/L)	75.67 ± 3.46	89.30 ± 19.05	84.00 ± 7.31	126.20 ± 39.83	74.40 ± 5.73	107.20 ± 15.45
ALT (U/L)	10.67 ± 0.88	14.71 ± 0.92	12.71 ± 1.94	20.50 ± 6.46	15.29 ± 3.91	22.67 ± 2.03
Total cholesterol (mg/dL)	179.80 ± 11.70	102.10 ± 13.86	171.30 ± 13.30	131.20 ± 20.78	155.50 ± 12.33	121.10 ± 8.48
Triglycerides (mg/dL)	136.20 ± 8.54	151.20 ± 18.60	156.90 ± 8.56	159.80 ± 10.08	109.70 ± 7.68	112.60 ± 8.15
Total protein (g/dL)	6.00 ± 0.13	5.20 ± 0.17	5.88 ± 0.21	4.98 ± 0.23	5.83 ± 0.21	5.70 ± 0.09
Albumin (g/dL)	3.83 ± 0.13	3.24 ± 0.13	3.64 ± 0.18	3.40 ± 0.14	3.53 ± 0.18	4.02 ± 0.14*
HDL (U/L)	211.80 ± 10.89	193.40 ± 30.46	207.70 ± 13.22	279.80 ± 81.40	255.92 ± 28.87	260.80 ± 27.01

BUN: blood urea nitrogen, AST: aspartate aminotransferase, ALT: alanine aminotransferase, and HDL: lactate dehydrogenase. Values represent the mean ± SEM (*n* = 10/group). *Statistically different from the control group (ANOVA followed by Dunnett's test, *P* < 0.05).

**Table 4:** Effect of the essential oil of *Hyptis martiusii* (EOHM, 100 and 500 mg/kg, p.o.) on absolute (g) and relative organ weight (g/10 g of animal body weight) in male and female Swiss mice treated orally for 30 days.

Parameter	Control		EOHM 100 mg/kg		EOHM 500 mg/kg	
	Male	Female	Male	Female	Male	Female
Heart (g)	0.25 ± 0.01	0.18 ± 0.00	0.23 ± 0.01	0.18 ± 0.01	0.22 ± 0.01	0.15 ± 0.00
(g/10 g)	0.05 ± 0.00	0.05 ± 0.05	0.05 ± 0.00	0.04 ± 0.00	0.05 ± 0.00	0.04 ± 0.00
Lung (g)	0.24 ± 0.02	0.24 ± 0.04	0.26 ± 0.02	0.28 ± 0.03	0.23 ± 0.02	0.22 ± 0.00
(g/10 g)	0.05 ± 0.00	0.05 ± 0.00	0.06 ± 0.00	0.05 ± 0.00	0.05 ± 0.00	0.06 ± 0.02
Liver (g)	2.21 ± 0.31	2.11 ± 0.10	2.03 ± 0.19	2.00 ± 0.05	2.22 ± 0.14	1.92 ± 0.06
(g/10 g)	0.48 ± 0.06	0.46 ± 0.02	0.44 ± 0.04	0.42 ± 0.03	0.53 ± 0.03	0.54 ± 0.01
Kidney (g)	0.33 ± 0.02	0.23 ± 0.01	0.34 ± 0.02	0.25 ± 0.01	0.30 ± 0.01	0.22 ± 0.00
(g/10 g)	0.07 ± 0.00	0.05 ± 0.00	0.07 ± 0.00	0.05 ± 0.00	0.07 ± 0.00	0.06 ± 0.02*
Adrenal (g)	0.02 ± 0.00	0.01 ± 0.00	0.01 ± 0.00	0.01 ± 0.00	0.01 ± 0.00	0.01 ± 0.00
(g/10 g)	0.004 ± 0.001	0.005 ± 0.003	0.003 ± 0.001	0.002 ± 0.000	0.002 ± 0.000	0.002 ± 0.004
Spleen (g)	0.28 ± 0.08	0.33 ± 0.03	0.21 ± 0.04	0.27 ± 0.041	0.20 ± 0.03	0.25 ± 0.02
(g/10 g)	0.06 ± 0.02	0.07 ± 0.00	0.04 ± 0.01	0.05 ± 0.01*	0.05 ± 0.01	0.07 ± 0.00
Stomach (g)	0.26 ± 0.04	0.28 ± 0.02	0.30 ± 0.03	0.29 ± 0.02	0.25 ± 0.02	0.30 ± 0.02
(g/10 g)	0.05 ± 0.00	0.06 ± 0.00	0.06 ± 0.00	0.07 ± 0.00	0.06 ± 0.00	0.08 ± 0.00
Intestine (g)	0.26 ± 0.02	0.21 ± 0.01	0.23 ± 0.02	0.21 ± 0.01	0.22 ± 0.04	0.20 ± 0.02
(g/10 g)	0.05 ± 0.01	0.14 ± 0.09	0.05 ± 0.00	0.05 ± 0.00	0.05 ± 0.01	0.05 ± 0.00
Pancreas (g)	0.29 ± 0.07	0.43 ± 0.08	0.34 ± 0.07	0.38 ± 0.04	0.32 ± 0.7	0.431 ± 0.03
(g/10 g)	0.06 ± 0.01	0.22 ± 0.13	0.07 ± 0.01	0.08 ± 0.01	0.08 ± 0.02	0.12 ± 0.01
Brain (g)	0.39 ± 0.00	0.45 ± 0.01	0.44 ± 0.01	0.46 ± 0.01	0.39 ± 0.01	0.43 ± 0.01
(g/10 g)	0.09 ± 0.00	0.09 ± 0.00	0.09 ± 0.00	0.10 ± 0.00	0.09 ± 0.00	0.12 ± 0.00
Testicle (g)	0.09 ± 0.02	—	0.11 ± 0.01	—	0.10 ± 0.00	—
(g/10 g)	0.02 ± 0.00		0.02 ± 0.00		0.02 ± 0.00	
Prostate (g)	0.01 ± 0.00	—	0.01 ± 0.00	—	0.01 ± 0.00	—
(g/10 g)	0		0		0	
Uterus (g)	—	0.09 ± 0.02	—	0.15 ± 0.03	—	0.10 ± 0.01
(g/10 g)		0.04 ± 0.02		0.03 ± 0.01		0.03 ± 0.04
Ovary (g)	—	0.02 ± 0.00	—	0.08 ± 0.05	—	0.13 ± 0.00
(g/10 g)		0.01 ± 0.00		0.02 ± 0.01		0.03 ± 0.00

Values represent the mean ± SEM (*n* = 5/group). *Statistically different from the control group (ANOVA followed by Dunnett's test, *P* < 0.05).
